# Emotion regulation, academic buoyancy, and academic adjustment of university students within a self-determination theory framework: A systematic review

**DOI:** 10.3389/fpsyg.2022.1057697

**Published:** 2022-11-29

**Authors:** Marina Kritikou, Theodoros Giovazolias

**Affiliations:** Department of Psychology, University of Crete, Crete, Greece

**Keywords:** emotion regulation, academic buoyancy, self-determination theory, students, systematic review

## Abstract

**Introduction:**

The transition from secondary to tertiary education seems to be a stressful period for many students since they need to adjust to the new academic environment.

**Method:**

This article is a systematic review of 4,285 articles. The aim of this review was to investigate the factors in the university environment associated with emotion regulation, academic buoyancy, and academic adjustment of tertiary students within a self-determination theory framework in combination with the nascent third wave of Positive Psychology. Forty-one articles met the inclusion criteria, all of which were rated as either good or moderate quality.

**Results:**

The bulk of the studies included in the systematic review reported individual factors, i.e., self-efficacy, intrinsic motivation, academic factors, i.e., intention to drop out, learning climate, and family and social factors i.e., faculty, peer, and parental autonomy support.

**Discussion:**

In accordance with the third wave of Positive Psychology that focuses on how interpersonal and ecological factors create nurturing environments and positive institutions, the systematic review highlighted the factors that institutes should consider in order to help students adjust better to the academic environment.

## Introduction

### Self-determination theory and positive psychology

#### Self-determination theory

Self-determination theory (SDT); (Ryan and Deci, 2000) is a macrotheory of human motivation, which addresses basic issues such as personality development, self-regulation, universal psychological needs, life goals and aspirations, the relationship of culture to motivation, and the impact of social environments on motivation, affect, behavior and wellbeing. SDT began by differentiating types of motivation and the initial idea was that the type or quality of a person's motivation would be more important than the total amount of motivation for predicting many important outcomes, such as psychological health and wellbeing (Elliot and Thrash, 2001; Deci and Ryan, 2008). The above hypothesis has been examined also in university students and was found that autonomous motivation positively predicted academic achievement, whereas controlled motivation predicted dropout intentions (Jeno et al., 2018; Corpus et al., 2020).

SDT examines a wide range of phenomena across gender, culture, age, and socioeconomic status and has 6 mini theories: cognitive evaluation theory, organismic integration theory, causality orientations theory, basic psychological needs theory, goal content theory, and relationships motivation theory (Deci and Ryan, 2015). Need satisfaction has been associated with wellbeing and healthy psychological development, whereas the frustration or even thwarting of basic psychological needs has been associated not only with ill-being but also with the pursuit of extrinsic life goals, i.e., materialism and fame (Kanat-Maymon et al., 2015; Vansteenkiste et al., 2020). According to large prospective research, relative intrinsic goals at baseline, i.e., meaningful relationships, community contributions, and personal growth, predicted experiencing greater need satisfaction and improved wellbeing over time (Hope et al., 2019). Moreover, the absence of need satisfaction does not necessarily imply the presence of need frustration, whereas the presence of need frustration denotes the absence of need satisfaction (Vansteenkinste and Ryan, 2013). Self-determination theory is an organismic theory, as it assumes that humans are active, working to integrate new material into their own sense of self; however, it also suggests that the environment can either provide nutrients for this integrative process or can disrupt and impair the process (Deci and Ryan, 2015).

#### Positive psychology: Third wave

Given the fact that mainstream psychology has focused primarily on disorder and dysfunction, the first wave of Positive Psychology (emerging around 1998/2000-2010) concentrated on the positive, i.e., positive phenomena including emotions, behaviors, cognitions, and organizations (Seligman and Csikszentmihalyi, 2000). The second wave (emerging around 2010–2015), although focused on flourishing and wellbeing, started focusing also on the dialectical nature of wellbeing and appreciating the ambivalent nature of the good life (Lomas and Ivtzan, 2015). The two waves are not mutually exclusive, but rather inform and complement each other (Lomas et al., 2021).

The third wave is a general movement of shifting from the individual toward greater complexity (Lomas et al., 2021; Wissing, 2022) and focuses on groups, organizations, and broader systems. This wave explores the multiple socio-cultural factors and processes that impact peoples' wellbeing by looking how various interpersonal and ecological factors can be better understood to create nurturing environments and positive institutions. It also puts greater emphasis on the empirical study of the above (Lomas et al., 2021).

In line with the third wave in Positive Psychology, SDT has already highlighted the learning environment is an important factor for the individual to flourish. An autonomy-supportive environment (i.e., perspective talking, demonstrating relevance, and providing opportunities for choice and self-regulation) has been proposed as a key component to promoting a positive learning environment where students can thrive (Deci and Ryan, 2015). Research on university students has shown that supporting learners' autonomy, providing choices and options, determining and acknowledging student perspectives, and trying to understand their viewpoints significantly predicted wellbeing and basic psychological need satisfaction and frustration (Levesque et al., 2004; Basson and Rothmann, 2018; Neufeld, 2020).

### Emotion regulation

Emotions arise when something occurs and our body responds to this event behaviorally, experientially, or physiologically (Gross, 2002). According to Gross, a definition of emotion regulation defines the process by which we influence which emotions we experience, when we experience them, and how we experience and express them (Gross, 1998). It is more than decreasing negative emotions, as emotion regulation may also occur without conscious awareness and is neither inherently good nor bad (Gross, 2002). Emotion regulation changes across the life span, i.e., in infancy extrinsic emotion regulation is initially dominant, since caregivers play a major role, whereas in early to middle childhood, when developmental changes occur, additional emotion regulation capabilities are enabled. In addition, adolescence represents a developmental period with further changes as due to the maturation of prefrontal regions, new cognitive forms of emotion regulation are enabled (Gross, 2013). Gross (1998) proposed an information-processing model of emotion regulation that treats each step in the emotion-generative process as a potential target for regulation. At a later stage, Gross (2015) changed the information-processing model to an extended process model. This model pictured a process that unfolds over time in three consecutive stages: (i) identification of an emotional goal, (ii) selection of a strategy to regulate emotion (i.e., attentional deployment, cognitive reappraisal, etc.), and (iii) implementation of a particular tactic to regulate emotions (i.e., problem solving, visual distraction, meaning-making).

Within the SDT frame, emotions should be addressed as important sources of information, the awareness of which allows for greater autonomous regulation and enables the individual to unfold its potential and enhance its capacities for choice and authenticity (Vansteenkiste and Sheldon, 2006). The term *integrative emotion regulation* involves not only a non-critical, receptive attention to one's emotional experience but also an interested and volitional exploration of the above experience (Roth et al., 2019). Three forms of emotion regulation are proposed as follows: (a) integrative regulation that supports autonomy, (b) controlling regulation to direct reinterpret or minimize emotional inputs, and (c) dysregulation which emotions are poorly managed (Roth et al., 2009) and tested in university students. Relevant research has shown that integrative emotion regulation positively predicts wellbeing and mediates psychological needs satisfaction (Benita et al., 2019).

Combining Gross's model with the self-determination frame, a model was developed recently in which the role of autonomy experiences is considered within each stage (Benita et al., 2019). In the proposed model in the identification stage, the concept of autonomous vs. controlled reasons is included in order to pursue emotional goals. In the selection stage, the concept that has been proposed is that of emotion regulation styles, which are broader concepts, compared to emotion regulation strategies. As far as the implementation stage is concerned, apart from the particular tactics, the quality of implementation has been introduced (defensiveness vs. non-defensiveness and flexibility vs. rigidity) (Benita, 2020).

Studies conducted on university students examining the behavioral, emotional, and cognitive consequences of integrative emotion regulation and suppression of emotion, in relation to a fear-eliciting film have concluded that integrative regulation is associated with less defensive written expression (Roth et al., 2014). Furthermore, in relevant research, the relationship between integrative and suppressive emotion regulation and wellbeing was tested in three countries (Israel, Peru, and Brazil) and was found that integrative emotion regulation positively predicted wellbeing and was mediated by psychological need satisfaction in all three countries (Benita et al., 2019).

### Academic buoyancy

In order to differentiate academic buoyancy from academic resilience, Martin and Marsh (2008) reported that resilience has been characterized in terms of “acute” and “chronic” adversities that are seen as “major assaults” on the developmental process, whereas academic buoyancy reflects the ups and downs of everyday life as distinct from acute and chronic diversities. Academic buoyancy is associated with a more typical experience of poor performance, whereas academic resilience may be relevant to chronic underachievement (Martin and Marsh, 2008; Martin, 2013). Also, academic buoyancy is a distinct construct from that of adaptive coping, as it has been suggested that buoyancy is unrelated to coping; for example, an anxiety test explained a significant proportion of variance over and above that explained by coping (Putwain et al., 2012).

A number of motivational factors have been identified as being significantly associated with students' academic buoyancy: confidence (assessed *via* high self-efficacy), coordination (high planning), commitment (high persistence), composure (low anxiety), and control (low uncertain control). The above five motivational factors have been found to be significant predictors of academic buoyancy when subjected to longitudinal examination and also to partially mediate the effects of prior academic buoyancy on subsequent academic buoyancy (Martin et al., 2010).

### Aim and research questions

The aim of this review was to investigate the factors associated with emotion regulation, academic buoyancy, and academic adjustment of university students within a self-determination theory framework. In combination with the third wave of Positive Psychology, another aim was to examine the factors within the university environment that contributes to students' sense of academic buoyancy, emotion regulation, and academic adjustment.

The review questions are as follows:

- What factors are associated with HE students' emotion regulation according to SDT?
- What factors are associated with HE students' academic buoyancy according to SDT?
- What factors are associated with HE students' academic adjustment according to SDT?
- What factors are associated with basic need satisfaction and frustration in HE students according to SDT?

## Methods

### Search strategy

An advanced search was conducted by two independent reviewers. The search strategy was applied to Scopus, Web of Science, and PubMed. Gray literature was identified with Google Scholar. Databases were searched from March to May 2022.

The following term search was used: (a) Review Question 1: [(emotion regulation^*^ OR emotional control) AND (college students OR university students OR higher education students) AND (self-determination theory OR self determination theory)], (b) Review Question 2: [(college students OR university students OR higher education students) AND ( academic buoyancy) AND (self-determination theory OR self determination theory)], (c) Review Question 3: [(college students OR university students OR higher education students) AND (academic adaptation OR academic adjustment) AND (self-determination theory OR self determination theory)], and (d) Review Question 4: [(college students OR university students OR higher education students) AND ( basic psychological needs OR need frustration OR need satisfaction) AND (self-determination theory OR self determination theory)]. The search areas were the title, abstract, topic, and identifiers. In total, 4,285 articles were found and screened.

### Inclusion/exclusion criteria

**Figure 1:**
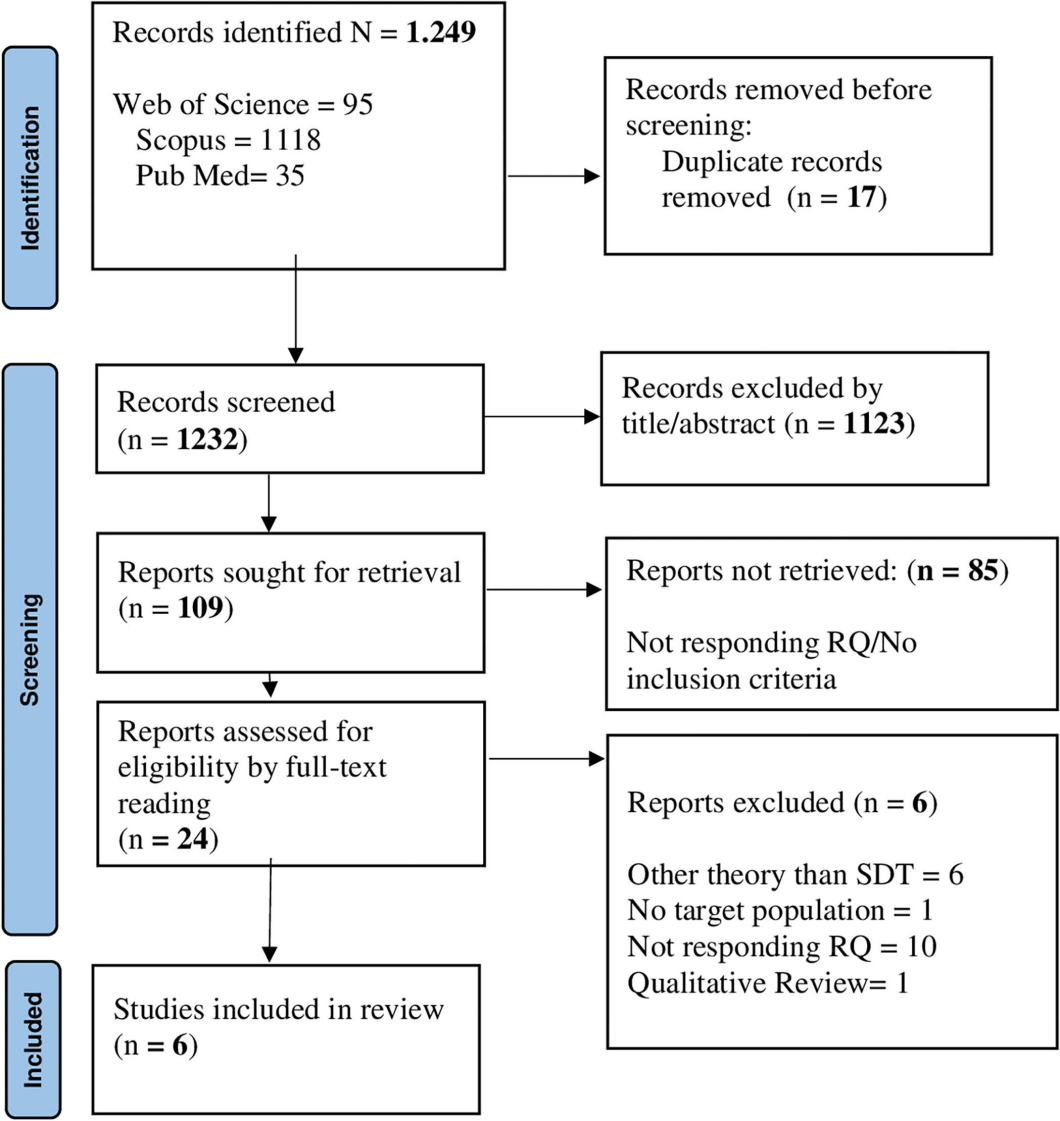
PRISMA flowchart for the factors are associated with HE students' emotion regulation.

**Figure 2:**
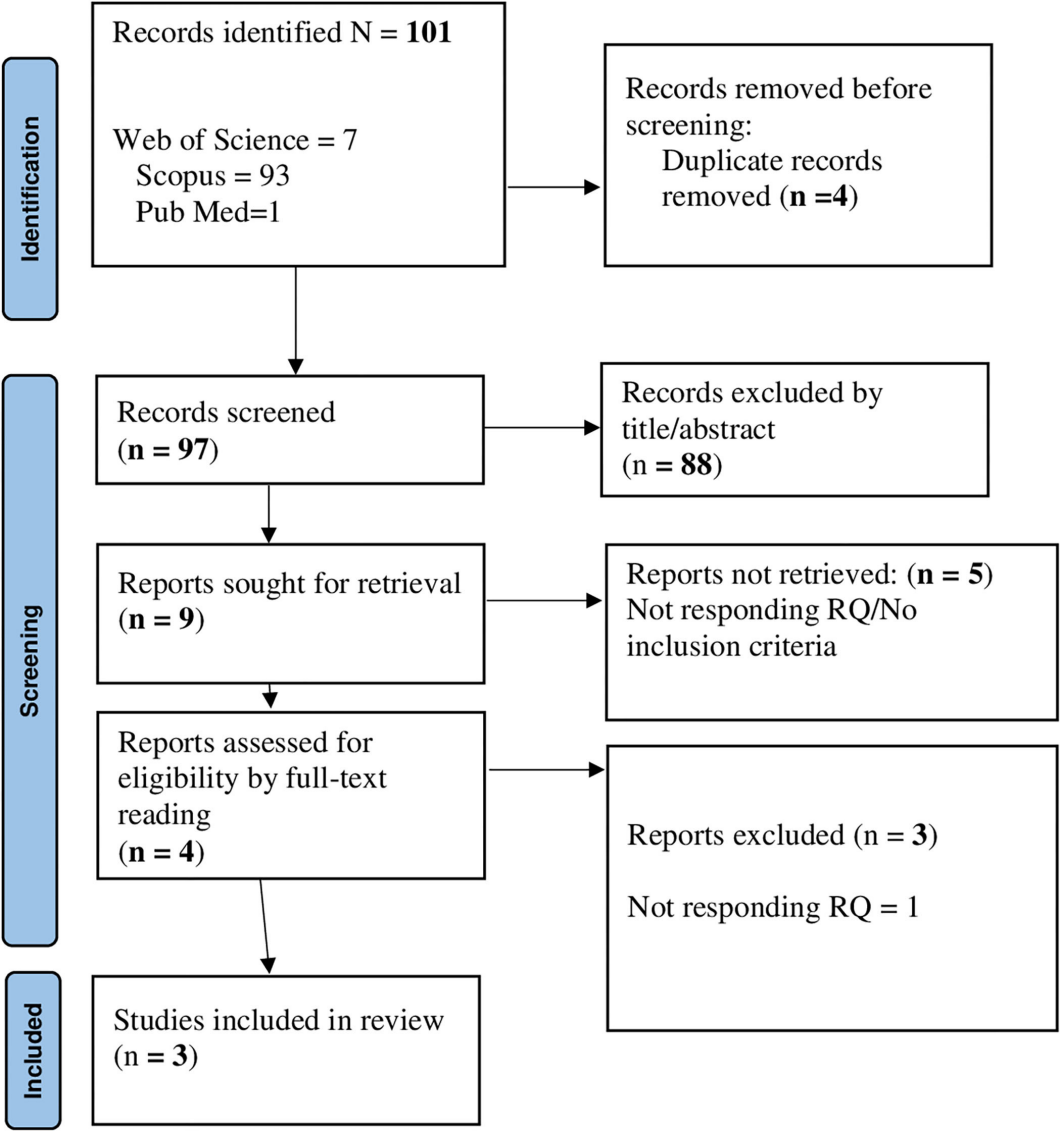
PRISMA flowchart for the factors are associated with HE students' academic buoyancy.

**Figure 3:**
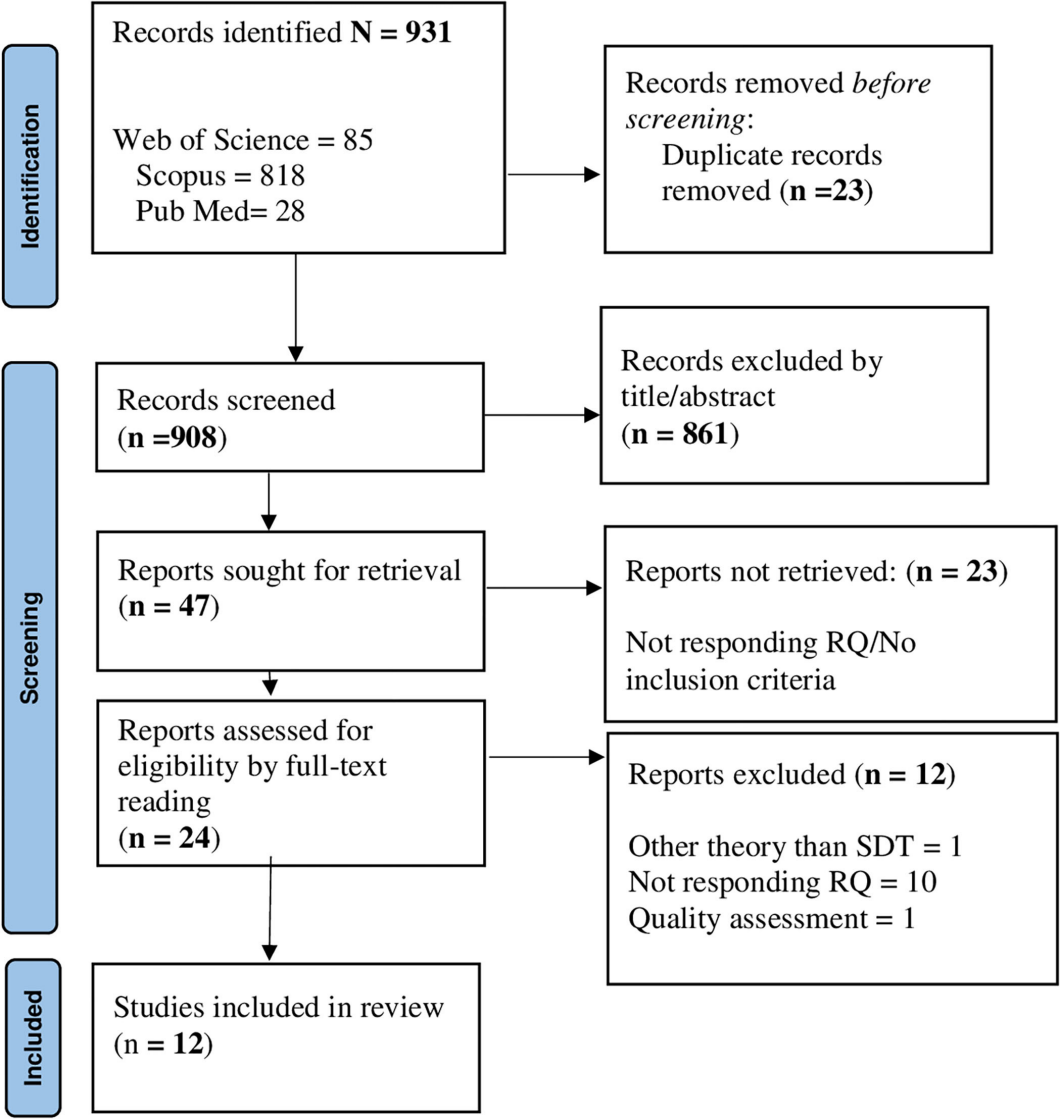
PRISMA flowchart for the factors are associated with HE students' academic adjustment.

**Figure 4:**
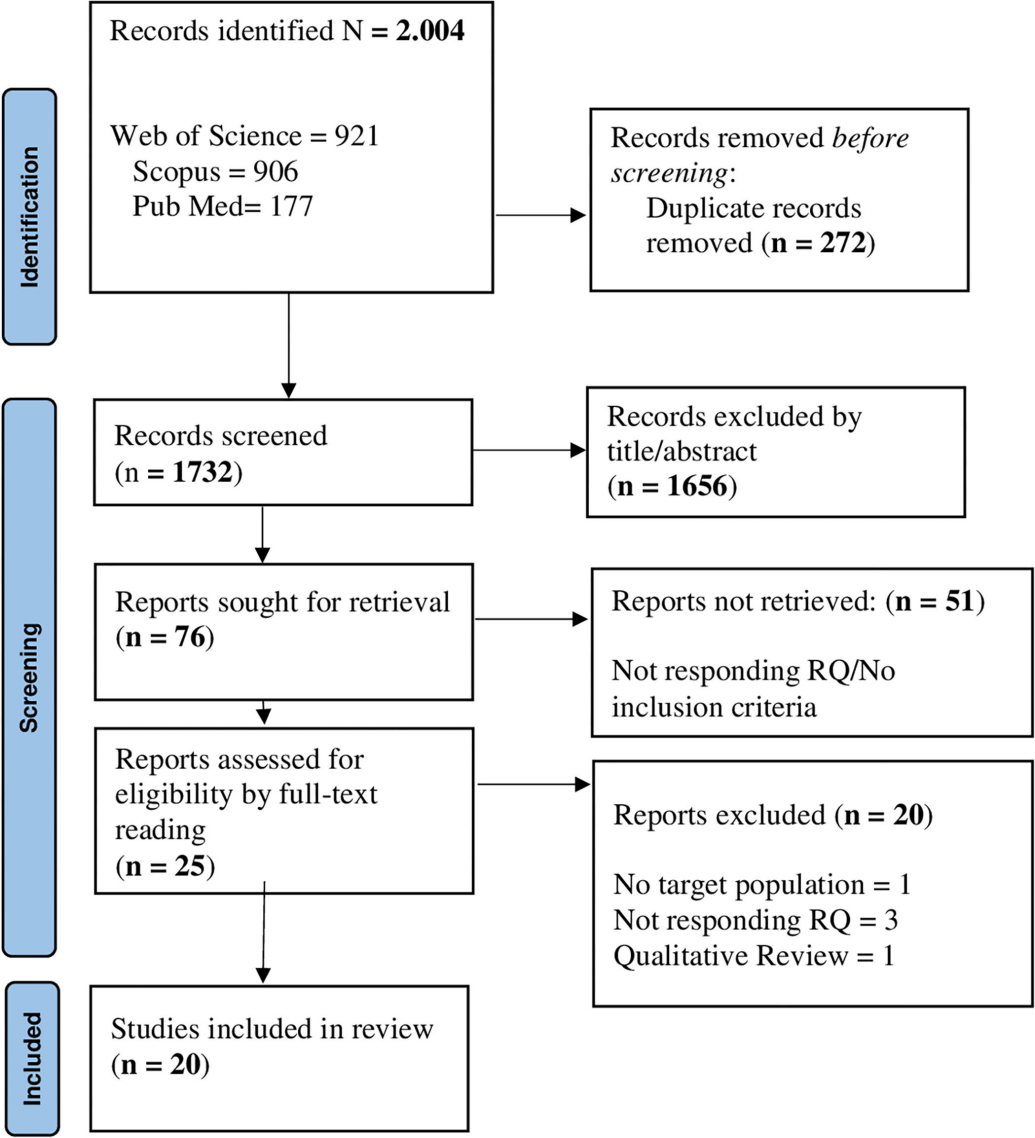
PRISMA flowchart for the factors are associated with HE students' basic need satisfaction and frustration.

An overview of our literature search and selection – based on the Preferred Reporting Items for Systematic Reviews and Meta-Analysis Protocols (PRISMA-P), (Moher et al., 2010) – is presented in Figures 1–4. The PRISMA-P is a guideline that consists of a 17-item checklist, intended to guide the development of protocols of systematic reviews and meta-analyses in order to answer a specific research question. It helps authors to describe the rationale and intended purpose of the review and the planned methodological and analytical approach. As it is suggested by Shamseer et al. (2015), a systematic review protocol is important as it allows systematic reviewers to plan and foresee possible problems; clearly document their steps before they begin the review process, enabling other researchers to compare and replicate the review methods if needed; prevents arbitrary inclusion and extraction criteria. Authors are generally encouraged to use PRISMA-P because of the lack of existing protocol guidance overall.

Regarding our study, by entering the search terms for the four review questions, we resulted in 1,249, 101,931, and 2,004 hits, respectively. To select appropriate studies, a number of inclusion and exclusion criteria were used. Studies were included if (a) participants were higher education students, (b) they employed quantitative, qualitative, or mixed-methods research methodology, (c) they contained a measure of emotion regulation, academic buoyancy, academic adjustment, or basic psychological needs as a dependent variable, (d) they were situated within a self-determination theoretical framework, and (e) they were published in an English-language peer-reviewed journal. Articles were excluded if: (a) they did not include higher education students or included students conducting a Masters or PhD degree, (b) included students having a mental illness diagnosis, (c) they were reported on a basis of other theoretical paradigms, or (d) they reported conference proceedings or did not present any empirical data.

### Data extraction

For each study/report, details concerning country and region, study aims, design, outcome of interest, sampling and recruitment, data collection methods, population characteristics, predictors and outcomes associated with students' sense of academic buoyancy, emotion regulation, academic adjustment and basic psychological needs satisfaction and frustration, ecological (institution's environment) risk and protective factors and top-line findings were extracted.

### Risk of bias

Quality assessment was performed following the Mixed-Methods Appraisal Tool (MMAT) version 2018 (Hong et al., 2018). This tool was developed for evaluating the methodological quality of empirical studies qualitative/quantitative/mixed methods. Two reviewers independently assigned the quality rating (range: 1–10). Studies reporting a score ≥5 were retrieved. Any discrepancy/disagreement was solved by discussions between the two reviewers.

### Procedure

The screening of articles took place in several phases. First, duplicates were removed, and then the two reviewers independently classified the abstracts for each review question as relevant or irrelevant. As a second step, the reviewers screened the articles in each review question by title or abstract. Based on the established exclusion and inclusion criteria, a vast majority of the studies were excluded. Afterward, the two reviewers independently checked for eligibility for the selected pool of articles. The remaining articles were assessed again for eligibility by full-text reading and quality assessment. The reviewers agreed on 90% of the ratings. Any discrepancies were discussed and resolved through discussion. Data appraisal and synthesis were performed narratively.

## Results

### Factors associated with HE students' emotion regulation according to SDT (RQ1)

For research question 1, 1,232 records were identified; of those, 1,123 were removed due to duplications. Next, 109 individual citations were screened by assessing the title/abstracts, and 85 records were removed since they were considered out of topic. After a careful evaluation of the remaining 24 articles, 18 records were eliminated, since they did not meet the inclusion criteria. Overall, 6 articles underwent full-text reading and 6 studies were included in the final analysis (Figure 1). The majority of the studies (*n* = 4) applied quantitative methods, and two (*n* = 2) studies applied a mixed approach. All records included in the final pool reached a quality score ≥5 in MMAT, indicating a satisfactory data collection, and coherence between data, analysis, and final interpretation (Supplementary Table 1).

According to SDT, and due to the fact that only a few studies met the criteria for research question 1, the predictors associated with higher education (HE) students' emotion regulation were gratitude and kindness, the three basic psychological needs (competence, relatedness, and autonomy), the difficulty to perform purposeful behaviors related to emotions, and the emotion creativity.

As for the first factor, gratitude and kindness, in an online pilot intervention (Datu et al., 2021), participants in the kindness and gratitude group, had significantly higher scores for positive emotions, than those assigned in the control condition. Furthermore, the three basic psychological needs (competence, relatedness, and autonomy) seemed to be the predictors of students' emotion regulation (Holzer et al., 2021). Other factors associated with students' emotion regulation were the autonomous motivation cluster since the more self-determined types of motivation were positively associated with pleasant emotions and achievement (Gonzalez et al., 2012) and the difficulty to perform purposeful behaviors related to emotions (Bytamar et al., 2020). Finally, other factors were emotion creativity, which was associated with the positive emotions of gratitude, hope, and love (Oriol et al., 2016), and emotion regulation style with integrative emotion regulation was associated with less defensive processing of negative experiences and better functioning (Roth et al., 2018).

### Factors associated with HE students' academic buoyancy according to SDT (RQ2)

For research question 2, 101 records were identified; of those, 4 were removed due to duplications. Next, 97 individual citations were screened by assessing the title/abstracts, and 88 records were removed since they were considered out of topic. After a careful evaluation of the remaining 9 articles, 5 records were eliminated, since they did not meet the inclusion criteria. Overall, 4 articles underwent full-text reading and 3 studies were included in the final analysis (Figure 2). All studies (*n* = 3) applied quantitative methods. All records included in the final pool reached a quality score ≥5 in MMAT, indicating a satisfactory data collection, and coherence between data, analysis, and final interpretation (Supplementary Table 2).

According to SDT, three factors associated with higher education (HE) students' academic buoyancy were identified. Autonomous motivation was the first factor that was found to be positively associated with academic buoyancy (Aydin and Michou, 2019). Also, students' adaptability was another significant predictor of students' university academic achievement, beyond the effects of buoyancy and motivation (Holliman et al., 2019). In addition, personal best goals were found to be positively and significantly associated with academic buoyancy, and thus, adapting personal best goals can be a technique to achieve students' everyday resilience (Jahedizadeh et al., 2021).

### Factors associated with HE students' academic adjustment according to SDT (RQ3)

For research question 3, 931 records were identified; of those, 28 were removed due to duplications. Next, 908 individual citations were screened by assessing the title/abstracts, and 861 records were removed since they were considered out of topic. After a careful evaluation of the remaining 47 articles, 23 records were eliminated, since they did not meet the inclusion criteria. Overall, 24 articles underwent full-text reading and 12 studies were included in the final analysis (Figure 3). All studies (*n* = 12) applied quantitative methods. All records included in the final pool reached a quality score ≥5 in MMAT, indicating a satisfactory data collection, and coherence between data, analysis, and final interpretation (Supplementary Table 3).

According to SDT, factors associated with higher education (HE) students' academic adjustment were identified, critically appraised, and summarized as follows: academic, individual, and family factors. What is more, as far as the institutional environment is concerned, the factor that was found to be protective for students' academic adjustment was perceived autonomy from teachers, a factor that significantly predicted autonomous motivation and self-efficacy (Girelli et al., 2018).

As for academic factors, variables that were found to be associated with students' academic adjustment were learning engagement and goal orientations (Wang et al., 2021), intention to drop out and academic self-efficacy (Girelli et al., 2018), and high school GPA and obtained credit points (van der Zanden et al., 2019). Considering individual factors, several other variables were found to be associated with students' academic adjustment. Firstly, academic motivation was found to be an associated variable in a few types of research (e.g., Levpuscek and Podlesek, 2019; Noyens et al., 2019). In addition, motivation variables, i.e., autonomous motivation (Miquelon et al., 2005; Bailey and Phillips, 2015; Willems et al., 2021), self-oriented perfectionism (Miquelon et al., 2005), self-efficacy, and learning strategies (Willems et al., 2021), as well as basic psychological needs satisfaction were also found to be associated with students' academic adjustment (Carr et al., 2013; Vergara-Morales and Del Valle, 2021). In terms of family factors, parental autonomy support (Daniels et al., 2019), parental involvement (Smojver-Ajic et al., 2015), and attachment styles were significantly linked to attachment security (positive relationship with the insecurity dimension) (Carr et al., 2013).

### Factors associated with basic need satisfaction and frustration in HE students according to SDT (RQ4)

For research question 4, 2,004 records were identified; of those, 272 were removed due to duplications. Next, 1,732 individual citations were screened by assessing the title/abstracts, and 1,656 records were removed since they were considered out of topic. After a careful evaluation of the remaining 76 articles, 51 records were eliminated, since they did not meet the inclusion criteria. Overall, 25 articles underwent full-text reading, and 20 studies were included in the final analysis (Figure 4). All studies (*n* = 20) applied quantitative methods. All records included in the final pool reached a quality score ≥5 in MMAT, indicating a satisfactory data collection, and coherence between data, analysis, and final interpretation (Supplementary Table 4).

According to SDT, factors associated with higher education (HE) students' basic need satisfaction and frustration were identified, critically appraised, and summarized as follows: academic, individual, and family–social factors. In terms of academic factors, academic satisfaction and experiences of autonomy support in the learning environment (Jeno et al., 2018; Schenkfenfelder et al., 2020; Yu and Levesque-Bristol, 2020), learning climate (Orsini et al., 2012; Neufeld, 2020), academic adjustment (Levesque et al., 2004; Law and Liu, 2021), and college adjustment, as well as study commitment (Hagenauer et al., 2017; Babenko et al., 2018; Benlahcene et al., 2020) were variables that predicted students' satisfaction needs. As for individual factors, life satisfaction, resilience, and wellbeing (Levesque et al., 2004; Vansteenkiste et al., 2016; Gunnell et al., 2017; Hagenauer et al., 2017; Neufeld, 2020), study commitment and engagement (Sulea et al., 2015; Hagenauer et al., 2017), self-efficacy and intimacy (Faye and Sharpe, 2008; Wood and Macakova, 2022) seemed to be related to students' needs. Finally, faculty and peer support were recognized as protective factors for students' psychological needs and frustration (Basson and Rothmann, 2018; Schenkfenfelder et al., 2020).

Furthermore, as far as the institutional environment is concerned, several factors were found to be protective for the students' psychological needs satisfaction. At first, the relationship between student and advisor and the opportunity to connect with faculty members were related to academic satisfaction and volitional autonomy (Schenkfenfelder et al., 2020). In addition, personalized feedback acknowledged feelings and interpersonal involvement predicted psychological needs satisfaction (Levesque et al., 2004; Basson and Rothmann, 2018). Further, learner's autonomy, i.e., providing choices and options, acknowledging student perspectives, and trying to understand their viewpoints, seemed to be important factors in order for the institution to be supportive to the students' needs (Orsini et al., 2012; Neufeld and Malin, 2019; Neufeld, 2020; Yu and Levesque-Bristol, 2020). Finally, institutions in which students were included in interdisciplinary first-year projects, were engaged actively in their learning process, generated ideas, and improved general skills such as conflict management, negotiation, and communication skills (Koch et al., 2016).

Hindering students' autonomy was identified as a risk factor for students' psychological needs satisfaction considering the institutional environment, i.e., giving directives or commands, using controlling language, providing answers, over-praising and spoon-feeding, being dismissive and/or defensive, being unaware of curriculum, unfair judgment, not providing relevance of content or teaching, using incentives (rewards and punishments) to motivate students (Neufeld and Malin, 2019; Neufeld, 2020).

## Discussion

### Summary of the main findings

On the one hand, the results of the narrative synthesis highlighted the fact that only a few studies have examined emotion regulation and academic buoyancy within a self-determination framework. On the other hand, as for the factors associated with university students' academic adjustment and basic need satisfaction and frustration, a greater number of studies were found suitable according to the inclusion and exclusion criteria.

Summarizing the results for review question 1, only a few factors were associated with higher education (HE) students' emotion regulation. These factors seemed to be gratitude and kindness, the three basic psychological needs (competence, relatedness, and autonomy), the difficulty to perform purposeful behaviors related to emotions, and the emotion creativity. These factors are in line with another review that highlighted affective, cognitive, motivational, and individual factors to be associated with emotion regulation (Matthews et al., 2021). A possible explanation for the fact that few articles were found to be eligible could be the fact that based on the literature described in the introduction (Roth et al., 2014, 2019; Benita et al., 2019; Benita, 2020), the field of emotion regulation was only recently developed within the self-determination framework. Another reason could be the fact that the research literature has focused more on emotion regulation among children or adolescents and less on emerging adulthood according to a previous review (Rawana et al., 2014). As for review question 2, only a few studies that examined academic buoyancy in university students were included. The above studies showed autonomous motivation, students' adaptability, and personal best goals to be positively and significantly associated with academic buoyancy. Indeed, empirical studies have suggested that when students are not able to effectively navigate the typical difficulties and challenges in their educational setting (as demonstrated by low buoyancy); this disrupts adaptive patterns of motivation and engagement (e.g., Martin et al., 2013). Other researchers (e.g., Collie et al., 2015) have also stressed that academic buoyancy is associated with an internal locus of control over academic outcomes that are an important element for their motivation and engagement.

However, as the majority of the research conducted in this field concerns elementary or high school educational settings (Martin and Marsh, 2008; Martin et al., 2010; Devi et al., 2019), there is a need for further research on the role and the associated factors of academic buoyancy in the tertiary education.

As far as questions 3 and 4 are concerned, factors associated with higher education (HE) students' academic adjustment and basic psychological need satisfaction were classified into academic, individual, and family factors. These factors that we found to be relevant also emerged from a recent review study (Zak-Moskal and Garrison, 2020), which highlighted the correlation between student retention and the college's failure to meet students' basic psychological needs. What is more, perceived autonomy from teachers was found to be protective for students' academic adjustment as far as the institutional environment is concerned. Lastly, the relationship between student and advisor and the opportunity to connect with faculty members, personalized feedback, acknowledged feelings, interpersonal involvement, and learner's autonomy have been found to predict the satisfaction of psychological needs (Lyness et al., 2013).

### Limitations and future research directions

From a methodological point of view, access to all databases was inevitable; therefore, some research have not been included in the quality assessment. Moreover, according to the review's inclusion criteria, only undergraduate students were involved, so future research should also consider Master or PhD students and how they adjust to the academic demands. What is more, focus should be given to students that cope with mental illnesses during their studies.

## Conclusion

This review tried to investigate the factors in the university environment that are associated with emotion regulation, academic buoyancy, and academic adjustment of tertiary students within a self-determination theory framework in combination with the nascent third wave of Positive Psychology. In line with the third wave in Positive Psychology, SDT has already highlighted the importance of the learning environment as an important factor for the individual to flourish. An autonomy-supportive environment (i.e., perspective talking, demonstrating relevance, and providing opportunities for choice and self-regulation) was identified as a key component to promoting a positive learning environment where students can thrive. Academic institutions need to prepare for today's students and help them to engage academically throughout their studies; feeling supported and having optimal learning experiences during their academic life is meaningful, life-enhancing, and resonates in students' later lives.

Practitioners working in the field of student counseling could create prevention programs emphasizing in cultivating the skills identified as associating factors for emotion regulation, academic buoyancy, academic adjustment, and basic psychological needs satisfaction and frustration (Hui and Tsang, 2012). Helping university students see the value in the activities, as well as acknowledging any potential difficulties could increase their motivation and commitment to their studies (Graham and Vaughan, 2022). Lastly, increasing students' academic buoyancy could help them deal with academic pressures and challenges and solve problems effectively facing at their academic settings (Absellatif, 2022).

## Data availability statement

The original contributions presented in the study are included in the article/Supplementary material, further inquiries can be directed to the corresponding author/s.

## Author contributions

MK and TG contributed to research design, literature review, and writing up of the manuscript. All authors contributed to the article and approved the submitted version.

## Conflict of interest

The authors declare that the research was conducted in the absence of any commercial or financial relationships that could be construed as a potential conflict of interest.

## Publisher's note

All claims expressed in this article are solely those of the authors and do not necessarily represent those of their affiliated organizations, or those of the publisher, the editors and the reviewers. Any product that may be evaluated in this article, or claim that may be made by its manufacturer, is not guaranteed or endorsed by the publisher.
